# The Proposed Registration of Nurses

**Published:** 1889-12-28

**Authors:** 


### THE PROPOSED REGISTRATION OF NURSES.

We are obliged to hold over a further instalment of the signatures to the memorial of the nurse training schools and institutions against the proposals of the British Nurses' Association. We may mention, however, that signatures to the protest are still coming in. Names may be sent to Dr. Steele, Guy's Hospital, St. Thomas-street, London, S.E.

